# Visual analysis of sorrel (Rumex spp.) genotypes by the biplot method using morphological characters

**DOI:** 10.18699/vjgb-26-98

**Published:** 2026-10

**Authors:** N. Sabaghnia, M. Mohebodini, M. Janmohammadi, M. Erfani, K. Farmanpour-Kalalagh

**Affiliations:** Department of Plant Production and Genetics, Faculty of Agriculture, University of Maragheh, Maragheh, Iran; Department of Horticultural Science, Faculty of Agricultural Science and Natural Resources, University of Mohaghegh Ardabili, Ardabil, Iran; Department of Plant Production and Genetics, Faculty of Agriculture, University of Maragheh, Maragheh, Iran; Department of Horticultural Science, Faculty of Agricultural Science and Natural Resources, University of Mohaghegh Ardabili, Ardabil, Iran; Department of Horticultural Science, Faculty of Agriculture, Tarbiat Modares University, Tehran, Iran

**Keywords:** dry leaf weight, discriminative capacity, perfect genotype, typical potential, сухая масса листьев, дискриминационная способность, идеальный генотип, типичный потенциал

## Abstract

Sorrel (Rumex spp.) belongs to Polygonaceae. It is distributed worldwide and used as vegetable crop and medicinal plant, so considerable research has been dedicated to its cultivation. Forty-four accessions from the Institut für Pflanzengenetik und Kulturpflanzenforschung (IPK), Germany, and ten genotypes collected from some areas of Iran were grown in two separate trials. The trials followed a completely randomized scheme with three replicates in a greenhouse environment. Seven traits were assessed: leaf length, blade length, lamina area, number of veins, petiole diameter, number of leaves, and dry leaf weight. The genotype × trait interaction (GTI) biplot model accounted for 77 and 78 % of the observed variability in the first and second trials, respectively. A nonagon and a heptagon were identified in trials, respectively, while genotypes 16 and 23 showed high mean values of dry leaf weight in the first and second trials, respectively. Genotypes 44 and 50 were identified as perfect genotypes, highly promising for sorrel genetic improvement programs, especially with regard to leaf yield. As for discriminative capacity, as well as typical potential, lamina area and petiole diameter best demonstrated differences among genotypes as perfect traits in both trials. There was a moderate positive correlation between dry leaf weight and lamina area across both trials (r = 0.57 for the first trial and r = 0.49 for the second trial). Therefore, lamina area can be considered an informative trait for constructing a selection index to improve leaf yield, along with dry leaf weight. The results demonstrate that lamina area and petiole diameter are key traits for selecting high-yielding sorrel genotypes, and the GTI biplot provides a practical visual approach to identify superior genotypes, facilitating efficient selection in breeding. Such a strategy can be recommended for future studies to describe prognostic and meticulous selection methods in order to recognize best traits for the breeding of sorrel and other vegetable crops.

## Abstract (ru)

Щавель (Rumex spp.) относится к семейству Polygonaceae, распространен по всему миру и используется как овощная культура и лекарственное растение, поэтому его культивирование стало предметом многочисленных исследований. В двух отдельных экспериментах были выращены 44 образца из Института генетики растений и исследований сельскохозяйственных культур (IPK, Германия) и 10 генотипов, собранных в разных районах Ирана. Эксперименты проводились в тепличных условиях по полностью рандомизированной схеме в трех повторениях, в ходе которых оценивались семь признаков: длина листа, длина листовой пластинки, площадь листовой пластинки, количество жилок, диаметр черешка, количество листьев и сухая масса листа. Оценка взаимодействия генотипа и признака (GTI) методом биплот-анализа объяснила 77 и 78 % наблюдаемой изменчивости в первом и втором экспериментах соответственно. В ходе испытаний были выявлены два типа расположения генотипов: нонагональное в первом и гептагональное во втором испытании, при этом генотипы 16 и 23 показали высокие средние значения сухой массы листьев в первом и втором испытаниях соответственно. Генотипы 44 и 50 были определены как идеальные, имеющие решающее значение для программы генетического улучшения щавеля, особенно с точки зрения урожайности листьев. Что касается дискриминационной способности, а также типичного потенциала, площадь листовой пластинки и диаметр черешка позволили выявить различия между генотипами как идеальными признаками в обоих испытаниях. Между сухой массой листьев и площадью листовой пластинки в обоих экспериментах наблюдалась умеренная положительная корреляция (r = 0.57 для первого эксперимента, r = 0.49 для второго эксперимента). Таким образом, площадь листовой пластинки можно считать информативным признаком для построения индекса отбора с целью повышения урожайности листьев, наряду с сухой массой. Результаты показывают, что площадь листовой пластинки и диаметр черешка являются ключевыми признаками для отбора высокоурожайных генотипов щавеля, а биплот-метод оценки GTI предоставляет практичный визуальный подход для выявления лучших генотипов, способствуя эффективному отбору в селекции. Подобная стратегия может быть рекомендована в будущих исследованиях для описания прогностических и тщательных методов отбора, которые помогут определить идеальные признаки для селекции щавеля и других овощных культур.

## Introduction

One of the ways to evaluate genetic diversity is the examination of morphological traits, which is relatively simple and inexpensive, so it is used to analyze genetic diversity in different crops. There are about 200 species of sorrel (Rumex spp.) around the world, and some of them are known as edible vegetables or traditionally used for medicinal purposes. The genus Rumex belongs to the Polygonaceae family, whose species are scattered all over the world. Many sorrel species have high economic value. They are used as both vegetables and medicinal plants, where roots, leaves, seeds, and herbal extract are generally utilized. Sorrel leaves are used as food in some areas (Li et al., 2022). Rumex rugosus Campd. is a stable plant, which has a branched stem and relatively small leaves. Its rapid spread in nature is due to the fact that buds appear on its underground and creeping stems. Rumex patientia L. is a perennial plant with very large, long, heart-shaped leaves with wavy edges and its root contains anthraquinone glucosides and tannin, whereas Rumex alpinus L. is a large and stable plant with a thick, heart-shaped leaves. Its raw leaves are eaten like spinach in salads, and while its petiole is used like asparagus (Vasas et al., 2015). Rumex pulcher L. is a biennial plant with leaves with a noticeable depression near the wide base, so the leaf has a velvet-like appearance. In the context of herbal medicine, its root has an appetizing effect. Rumex sculatus L. has relatively woody stems with thick bushes, and it is usually found on old walls, stony ground, ditches and similar places (Bourang et al., 2023). Rumex pseudonatronatus Murb. is a perennial herb native to Europe and Asia, often branching above the middle. It grows in wet and disturbed locations along lake shores, roadsides, fields, meadows. Rumex obtusifolius L. is a perennial herb up of 40 to 150 cm in height. It has large oval leaves, which can be eaten as salad or cooked like spinach (Mohebodini et al., 2020). Rumex kerneri Borbas is native to Europe and has been observed in some areas as an introduced species and roadside weed. It produces a slender erect stem from a thick taproot (Mohebodini et al., 2020).

The species of sorrel are rich in ascorbic acid and secondary metabolites: oxalic acid, phenolic acids, flavonoid compounds, phenolic compounds, anthraquinone, naphthalene, sterols and triterpenes, etc. (Vasas et al., 2015). The important anthraquinone emodin is produced in many species of this genus in their roots, seeds, leaves, stems, and flowers (Li et al., 2022). Therefore, it is important to identify and choose a suitable species or modify and introduce a variety of this valuable plant. In plant breeding, the first step is to select the most suitable individuals, so it is necessary first to collect and evaluate the genetic material and then apply proper breeding methods to it. Due to the importance of this plant, studies have been conducted in various fields to investigate its genetic diversity. While previous studies have examined the fruit morphology of sorrel species (Shahla et al., 2014), these traits alone are insufficient to distinguish taxa due to overlapping characteristics. Therefore, our study focuses on leaf morphological traits critical for evaluating the leaf yield potential and for selecting superior genotypes in breeding programs. The genetic variation of some genotypes of five Rumex species has been assessed using morphological traits, and the highest variation coefficients have been found in fresh and dry yield of leaves and in leaf number (Bourang et al., 2023). Also, the contents of phenols and antioxidants are higher in R. crispus than in other species, whereas R. tuberosus shows lower amounts in the measured morphologic and chemical characteristics in comparison to other species examined.

One of the most effective ways to assess genetic diversity involves the analysis of morphological traits, which is simple, cost-effective, and informative for plant breeding programs. Sorrel (Rumex spp.) comprises about 200 species worldwide, some of which are cultivated as leafy vegetables or used for medicinal purposes. Despite its economic and nutritional importance, there is limited knowledge regarding the morphological diversity of sorrel genotypes, particularly in relation to leaf yield performance. Identifying superior genotypes and key traits is crucial for improving leaf production and guiding breeding programs.

The objectives of this study were to: (1) evaluate genetic variation among 54 sorrel genotypes from international and local collections using leaf morphological traits, (2) assess genotype × trait interactions using the GTI biplot method, (3) identify best genotypes for leaf yield, and (4) determine the most informative traits for discriminating genotypes. This knowledge is essential for selecting promising genotypes and traits to enhance leaf yield in sorrel breeding programs under greenhouse conditions. This study aims to provide practical guidance for sorrel breeding by identifying superior genotypes and key morphological traits, thereby enabling breeders to make informed decisions and accelerate the development of high-yielding cultivars.

## Materials and methods

Trials, plant materials and traits. Two independent trials were performed to discover the interrelationships between leaf dry yield and some morphologic traits in 54 sorrel genotypes (Table 1), which had been acquired from the Institut für Pflanzengenetik und Kulturpflanzenforschung (IPK), Germany, or collected from some regions of Iran; as the latter could not be reliably identified to species, they are referred to as Rumex spp.

**Table 1.**
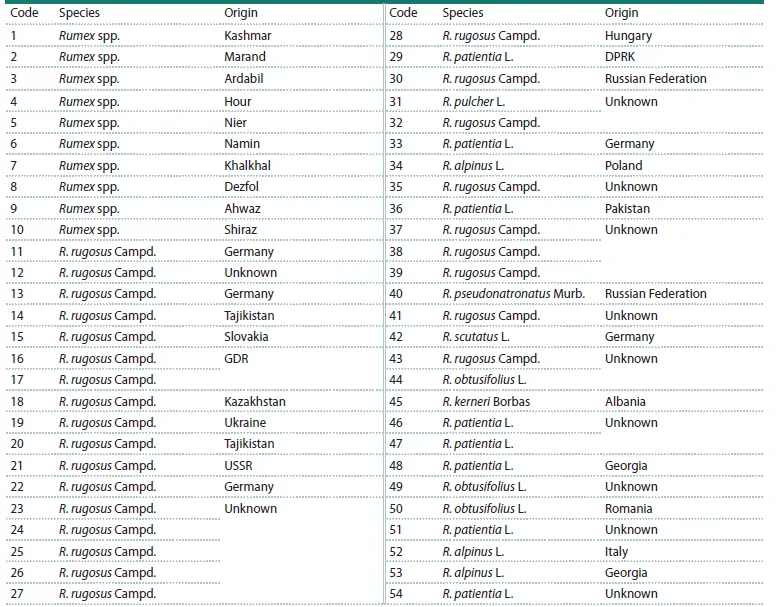
Codes and species types of sorrel genotypes (The first ten were collected in Iran, and the other were obtained from Genbank of the Leibniz Institute of Plant Genetics and Crop Plant Research, Germany) Note. GDR, German Democratic Republic; USSR, Union of Soviet Socialist Republics; DPRK, Democratic People’s Republic of Korea.

The study was centered on genotype × trait interactions, and all accessions, regardless of species, were included to capture the phenotypic diversity relevant to leaf yield improvement. These species were R. alpinus, R. kerneri, R. obtusifolius, R. patientia, R. pseudonatronatus, R. pulcher, R. rugosus, R. scutatus, and Rumex spp. Seeds of each genotype were sown individually in 30-cm diameter pots, one plant per pot. The experiment was arranged in a completely randomized design with three replications. Each genotype was represented by three plants per replication, providing sufficient replication for reliable estimation of morphological traits and leaf dry weight. Plants were grown under greenhouse conditions with a 12/12 h day/night schedule at 25/15 °C, using a 1:2:1 mixture of loamy soil, sand, and organic fertilizer. Irrigation and weeding were performed regularly throughout the growth period.

The morphologic traits were estimated as recommended by Mohebodini et al. (2020): leaf length (LL), blade length (BL), lamina area (LA), number of veins (NV), petiole diameter (PD), number of leaves (NL), and leaf dry weight (DW) with the use of a laboratory caliper, leaf area meter device, and lab scales. Leaf dry weight (DW) was measured by harvesting all leaves from each plant at the end of the growth period. The leaves were then oven-dried at 70 °C until complete desiccation and weighed. Focusing on leaf dry weight allows breeders to select genotypes that maximize the most valuable component for consumption and breeding purposes. Although leaf dry weight was the primary trait of interest, additional morphological traits were measured as component traits contributing to leaf biomass. Separate analyses were performed for each trial to account for minor differences in greenhouse conditions that might influence trait manifestation.

Statistical data analysis. The normality of the measured traits was assessed using the Anderson–Darling test. All traits in both trials showed no significant deviation from normality ( p > 0.05), confirming that the data were suitable for correlation analysis and subsequent GTI biplot modeling. Simple correlation coefficients between the traits were calculated using IBM-SPSS 25.0 (IBM-SPSS, Chicago, USA). For obtaining deeper insights into the interrelationships between traits and identifying those with the greatest impact on yield performance, the genotype × trait interaction (GTI) biplot model in the principal component analysis (PCA) layout was employed (Yan, Frégeau-Reid, 2018). The fitted model was generated with the GGEbiplot application, following the equation (Xu et al., 2017):

**Formula. 1.**
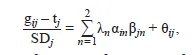
Formula1

where gij is the performance of genotype i for trait j, tj is the means of all gijs in trait j, SDj is the standard error for trait j, λn is the eigenvalue for PCAn, αin and βjn are the amounts for genotype i and trait j in PCAn, respectively, and θij is the magnitude of model residuals.

To obtain symmetrical values of genotypes and traits in the biplot, the eigenvalues (λ) of the principal components were adjusted using a scaling procedure to balance genotype and trait scores, enabling a clear graphical representation of the relationships among genotypes, traits, and their interactions. The transformed dataset was processed to enable clear graphical interpretation of GTI. Data were centered by trait mean (centering), scaled to make trait variations comparable (scaling), and analyzed using singular value decomposition (SVD) to obtain principal components. The scores for genotypes and traits were adjusted to produce a symmetric biplot, allowing accurate visualization of relationships among genotypes, traits, and their interactions. In the polygon and vector-view plots, circles represent ideal or reference points; the perfect genotype or perfect trait, derived from maximum observed trait values. Orange and green lines indicate the directions and magnitudes of trait vectors, connecting each genotype or trait to the origin for visualizing relationships and correlations. Genotypes or traits closest to these circles are considered optimal or most informative, respectively.

## Results and discussion

**Model fitting**

The PC1 and PC2 of the fitted GTI biplot model accounted for 60 to 17 % of the variation in the first trial and 60 to 18 % of the variation in the second trial within the datasets on sorrel (Fig. 1), indicating a substantial proportion of variation that demonstrated relationships among traits. Vectors were generated from the origin to the traits to explore such associations and to ease the explanations of interactions among traits (Yan, 2024). This fitted model pointed to the existence of the additive and crossover types of interaction, recommending that the rating of genotype in measured traits be altered. This is consistent with reports by Sabaghnia et al. (2015) on spinach and Sabaghnia et al. (2023) on garden cress, which emphasizes the complication of genotype selection without regarding GTI. In this research, the fitted biplot model, as suggested by Xu et al. (2017), proved to be successful for surveying and envisaging genotypes by trait associations. The fitted biplot model explained a remarkable magnitude of variation, with trait correlations revealed by the cosines of vectors.

**Fig. 1.**
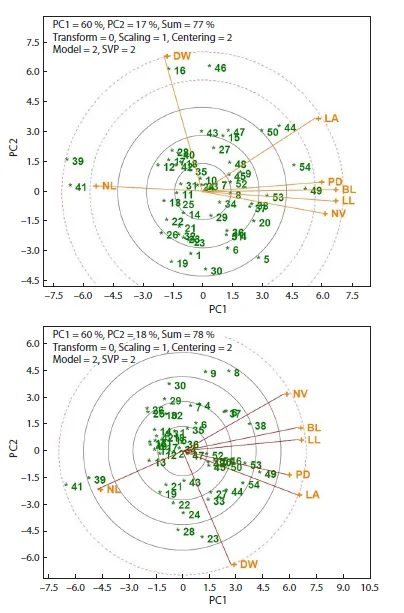
The vector diagram showing relationships among traits of sorrel in (upper panel) the first trial and (lower panel) the second trial.

**Vector-view analysis**

According to Figure 1, outstanding relationships included: (i) positive correlations among leaf length (LL), blade length (BL), petiole diameter (PD) and number of veins (NV); cosine 0° = +1; (ii) negative correlations of number of leaves (NL) with LL, BL, PD, and NV; cosine 180° = –1; (iii) near-zero association between leaf dry weight (DW) and number of leaves (NL); cosine 90° = 0; and (iv) near-zero association of leaf DW with LL, BL, PD and NV in the first trial. Also, outstanding relationships in the second trial included: (i) positive correlations between leaf length (LL) and blade length (BL); (ii) positive correlations between lamina area (LA) and petiole diameter (PD); (iii) negative correlations between number of leaves (NL) and number of veins (NV); (iv) near-zero association between leaf dry weight (DW) and number of leaves (NL); and (v) near-zero association between leaf dry weight (DW) and number of veins (NV). This agrees with findings by Mohebodini et al. (2020), who have shown positive correlations among leaf properties like leaf area and leaf length, as well as petiole diameter and number of veins, in various species of Rumex.

However, there was a moderate positive association between leaf dry weight (DW) with lamina area (LA) across both trials (Fig. 1), so lamina area should be considered in the determination of the selection index in sorrel breeding programs. Generally, the fitted biplot model provided an obvious visual indication of trait relations in sorrel, in agreement with the report by Bourang et al. (2023), who have found positive correlations between leaf dry weight and lamina area in some sorrel species. It should be noted that while the biplot method illustrates trait associations according to the structure of the dataset, correlation coefficients (Table 2) measure the direct interrelationship of traits and may not always directly align with the biplot results.

**Table 2.**
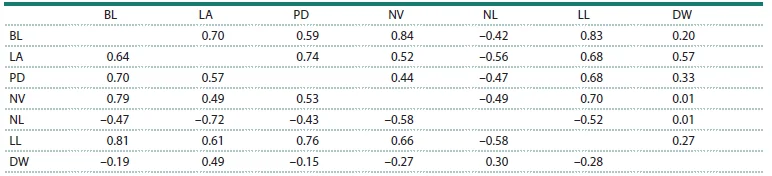
Correlations among traits of sorrels for (up diagonal) the first and (down diagonal) the second trial

**Polygon-view analysis**

Figure 2 demonstrates how the GTI biplot model illustrates genotypes and their comparison with regard to certain traits and detects those superior in specific sections, identifying them as candidates for using in breeding programs or releasing as commercial cultivars. A nonagon was identified in the first trial (Fig. 2), while the perpendicular lines to its sides ease comparison of the vertex genotypes. However, only four vertex genotypes showed the highest values of some traits; genotype 49 had the largest averages for leaf length (LL), blade length (BL), number of veins (NV) and petiole diameter (PD); genotype 44 showed the largest lamina area (LA); genotype 39 demonstrated the greatest number of leaves (NL); and genotype 16 was superior in mean leaf dry weight (DW) in the first trial. Conversely, five vertex genotypes (5, 19, 30, 41, and 46) did not show superior performance in any of the measured traits in the first trial.

**Fig. 2.**
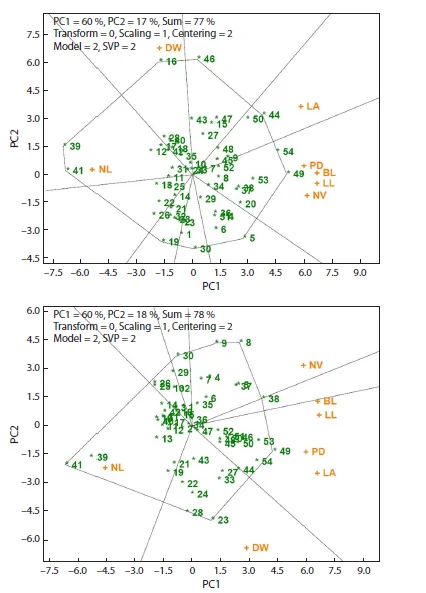
The nonagon (upper panel) and the heptagon (lower panel) showing relationships among traits and genotypes of sorrel in the first and second trials, respectively.

In the second trial, a heptagon was detected (Fig. 2), while only four vertex genotypes indicated the highest values for measured traits: genotype 49 had the large averages for leaf length (LL), blade length (BL), lamina area (LA) and petiole diameter (PD); genotype 41 showed a large number of leaves (NL); genotype 23 had a large leaf dry weight (DW); and genotype 8 showed a high mean for number of veins (NV) in the second trial. In contrast, three vertex genotypes (9, 30, and 38) did not manifest the most favorable performance in any of the traits in the second trial.

The benefits of the applied GTI biplot model are similar to those reported in other vegetable and field crops like quince (Rahimi et al., 2019) and safflower (Ebrahimi et al., 2023), showing that the method used in our study should be successful in extraction of useful outputs. The differing polygon shapes observed in the two trials – nonagon in the first and heptagon in the second – reflect the number of genotypes that were extreme for one or more traits in each trial. These differences likely result from minor variations in greenhouse conditions, which affected genotype performance and trait expression. This observation emphasizes that genotypes may respond differently to environmental conditions, even in controlled trials, and highlights the importance of multi-trial evaluation for reliable selection of superior genotypes.

**Perfect genotype position**

Figure 3 indicates the position of a perfect genotype, and the genotypes closest to this position are considered the best, so genotypes 44 and 50, followed by genotypes 47, 49, 53 and 54, were the closest to this position, indicating their superior performance in the first trial. Genotypes 39 and 41, although positioned farthest from the perfect genotype in the biplot, had the greatest number of leaves. However, this resulted in lower aggregate scores when considering multiple traits simultaneously. Hence, these genotypes can still be valuable in breeding programs, particularly for increasing leaf number in combination with traits such as lamina area and leaf dry weight.

**Fig. 3.**
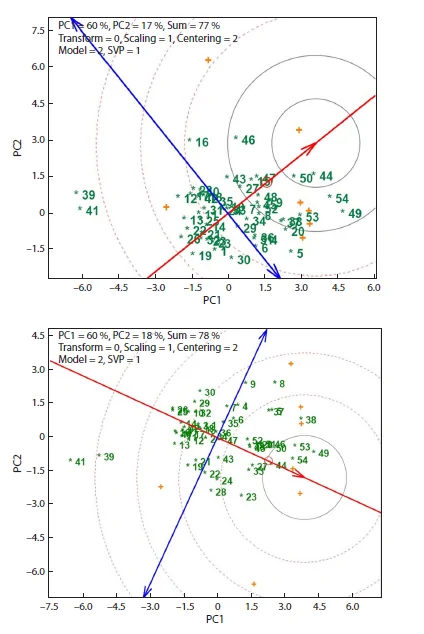
The perfect genotype diagram showing the positions of sorrel genotypes in (upper panel) the first trial and (lower panel) the second trial.

Similarly, in the second trial, genotypes 44, 49, 50, 53 and 54 were close to the perfect genotype (Fig. 3), showing their favorable performance, while genotypes 39 and 41 were located farthest from this position, regarding them less favorable in the second trial. These identified perfect genotypes are crucial for a sorrel genetic improvement program, especially since leaf yield performance and yield components often show low correlations in many vegetable crops (Shukla et al., 2016); thus, the employment of graphic multivariate models is vital for determining perfect genotypes and enhancing sorrel breeding strategies.

While leaf dry weight is the ultimate trait of interest for sorrel breeding, component traits such as lamina area, petiole diameter, and number of veins provide valuable information on leaf structure, expansion, and resource allocation. In some cases, genotypes with a large number of small leaves showed lower dry weight, reflecting the natural compromise between leaf size and leaf number. The GTI biplot enables simultaneous evaluation of multiple traits, capturing both additive and crossover interactions, and helps in the identification of genotypes with balanced performance across all important leaf characteristics. Although dry weight can be used alone for selection, the multivariate approach provides a more comprehensive assessment for breeding programs. Minor differences in greenhouse conditions between the two trials, such as temperature and light intensity, influenced trait expression, which justifies separate trial analyses. The observed differences in vertex genotypes across trials highlight genotype sensitivity to environmental variation and emphasize the importance of multi-trial evaluation to identify stable high-performing genotypes.

**Perfect trait position**

The discriminative capacity for a trait is the magnitude of the standard deviation that marks genotypes as different and can be shown by a perfect trait with such a hypothetical position (Fig. 4) that traits closer to this position have better discriminative capacities and farther traits are inferior in discriminative capacity. Based on the perfect trait biplot of the first trial, the following (in the descending order) traits were identified as most discriminative: lamina area (LA) followed by petiole diameter (PD), blade length (BL), leaf length (LL), and number of veins (NV). In contrast, traits such as number of leaves (NL) and leaf dry weight (DW) had low discriminative potentials in the first trial (Fig. 4); thus, they are not advised for future studies for distinguishing differences among sorrel genotypes.

**Fig. 4.**
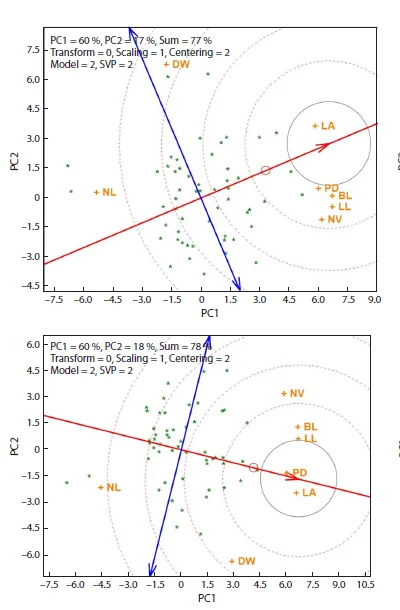
The perfect trait diagram showing position of the measured traits of sorrel in (upper panel) the first trial and (lower panel) the second trial

According to the perfect trait biplot of the second trial, lamina area (LA) and petiole diameter (PD), followed by blade length (BL) and leaf length (LL), were found to possess best discriminative capacities. Also, number of veins (NV) and leaf dry weight (DW) have a discriminative potential above average, indicating that they are effective in differentiating among sorrel genotypes in the second trial (Fig. 4). Finally, number of leaves (NL) had a low discriminative potential in the second trial, and it is not recommended for future investigations for revealing differences among sorrel genotypes.

The typical potential of a trait, showing its symbolic importance, is characterized by the angle between the trait’s vector and the axis of the average trait, smaller angles denoting greater typical potentials. Therefore, the traits with the smallest angles, such as lamina area (LA) and petiole diameter (PD), exhibit higher typical potential across both trials, while leaf dry weight (DW) had a larger angle with the axis, reflecting its lower typical potential in both trials.

**Analysis of leaf dry weight**

Figure 5 shows leaf dry weight (DW) along the horizontal axis, with distances from the axis reflecting variability among genotypes relative to the mean. Although accessions with the highest DW can be directly seen in Figure 2, Figure 5 highlights both mean performance and stability, assisting breeders in selecting genotypes with high and consistent leaf yield for further evaluation. Genotypes 16 and 46 were the most desirable for DW performance in the first trial, while genotypes 5 and 30 were the least favorable ones (Fig. 5). Genotypes with above-average performance of DW are situated above the average axis. The distance of genotypes from the horizontal axis serves as an index of standard deviation, and smaller distances indicate lower variability, so they are preferred for selection. Consequently, genotype 16 was recommended due to its low variability in comparison to genotype 30 in the first trial. In contrast, some genotypes located farther from the horizontal axis, such as 44 and 50, exhibit high variabilities and are considered less desirable.

**Fig. 5.**
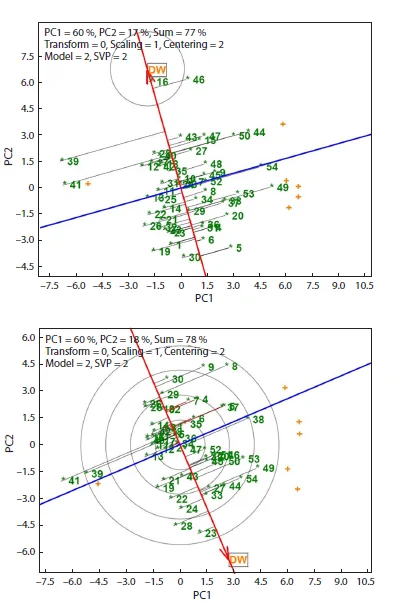
Examination of leaf dry weight (DW) across sorrel genotypes in (upper panel) the first trial and (lower panel) the second trial.

In the second trial, genotypes 23 and 28 were the most desirable for DW performance, while genotypes 9 and 30 were the least favorable ones (Fig. 5). The effectiveness of using this option for identifying favorable genotypes for target traits has been demonstrated in studies on sweet corn (Stansluos et al., 2023) and safflower (Ebrahimi et al., 2023).Sorrel genotype evaluation is crucial for preventing erosion in plant materials and effectively managing genetic improvement tasks. This study revealed substantial variation in leaf morphological traits among the 54 sorrel genotypes. While taxonomic identification was not available for all accessions, the observed phenotypic diversity provides useful information for selecting superior genotypes and identifying traits for breeding programs. These results should be interpreted as intrasample variation rather than interspecific diversity

The GTI biplot model revealed a relatively high level of variation (77 and 78 % for the first and second trials, respectively) and confirmed the presence of both simple and complex interactions among the genotypes and traits. Similar interaction patterns have been documented in other vegetable crops, such as spinach (Sabaghnia et al., 2015) and green bean (de Oliveira et al., 2018). These interactions resulted in varying rankings of genotypes across traits, making it challenging to select the most superior genotypes. Ignoring genotype × trait interactions can complicate the selection process, because genetic differences among genotypes lead to varying performance across traits. The GTI highlights these differences, enabling breeders to select genotypes with consistently desirable trait profiles

However, selection of high yielding genotypes needs genotypes with large lamina area, and it is similar in this respect to other leafy vegetable crops like spinach (Sabaghnia et al., 2013), so breeding for high leaf yield in sorrel should consider multiple traits, including lamina area, leaf number, and leaf dry weight (DW). Lamina area is particularly informative for selection because it moderately correlates with DW and can serve as a proxy at early steps of breeding. However, direct measurement of DW and consideration of leaf number are also important to ensure the development of high-yielding cultivars.

The GTI biplot proved effective for visualizing complex genotype × trait interactions, allowing breeders to identify superior genotypes and key traits for leaf yield, because this visual tool facilitates practical decision-making in breeding programs, enabling the selection of genotypes with optimal combinations of traits. While the present study focused on morphological traits of leaves to assess genetic diversity and leaf yield performance, we acknowledge that biochemical characteristics such as oxalate content, antioxidant capacity, and contents of other secondary metabolites are also important for evaluating the nutritional and medicinal value of sorrel. Integrating these biochemical traits in future studies could provide a more comprehensive assessment of genotype performance, allowing breeders to select genotypes that combine high leaf yield with superior nutritional or medicinal qualities. Such combined morphological and biochemical evaluations would further enhance the practical utility of the GTI biplot method in sorrel breeding programs.

## Conclusion

The study demonstrates the practical utility of the GTI biplot for sorrel breeding. By identifying lamina area as a key trait and highlighting superior genotypes such as 16, 23, 28, 46, 44, and 50, breeders can optimize breeding strategies and efficiently raise high-yielding cultivars. The GTI biplot explained 77 and 78 % of the variation in the first and second trials, respectively, demonstrating its utility for evaluating multivariate morphological data. Lamina area and petiole diameter were identified as the most discriminative and typical traits across both trials, providing key selection criteria for leaf yield improvement. However, for breeding programs aimed at industrial conditions, further evaluation in the field is recommended to ensure stability and performance in those target environments.

## Conflict of interest

The authors declare no conflict of interest.
